# Integrated Multi-Omics and Independent Validation Reveal MPO and TREM2 as Secretory Biomarkers for Non-Healing Diabetic Foot Ulcers

**DOI:** 10.3390/genes16121419

**Published:** 2025-11-28

**Authors:** Boya Li, Tianbo Li, Jiangning Wang, Lei Gao

**Affiliations:** Department of Orthopedics Surgery, Capital Medical University Affiliated Beijing Shijitan Hospital, No. 10 Yangfangdian Tieyi Road, Haidian District, Beijing 100038, China; liboya246@126.com (B.L.); litianbo3285@bjsjth.cn (T.L.); wangjn@bjsjth.cn (J.W.)

**Keywords:** diabetic foot ulcers, secretory protein-related biomarkers, machine learning, bioinformatics

## Abstract

**Background**: Diabetic foot ulcers (DFUs) are chronic wounds with high morbidity and mortality. Secretory proteins coordinate intercellular communication and may regulate inflammation, tissue repair and regeneration, but their contributions to DFU pathogenesis remain unclear. Aim: To discover and validate secretory protein–linked biomarkers that distinguish non-healing DFUs and to explore their potential utility for diagnosis and therapy. **Methods**: We integrated bulk RNA-sequencing datasets (GSE199939 training set; GSE80178 and GSE143735 validation sets) and a single-cell RNA-sequencing dataset (GSE223964). Differentially expressed genes, secretory protein lists, and weighted gene co-expression networks were intersected to select candidates. Functional enrichment, protein interaction networks and support vector machine–recursive feature elimination identified key markers. We visualized their cell-type distribution at single-cell resolution and validated their expression in external cohorts. Pathway enrichment, gene co-expression networks, ceRNA regulatory analysis and qRT-PCR in patient samples were used for further characterization. **Results**: Among 4803 differentially expressed genes, 743 overlapped with known secretory proteins. WGCNA highlighted modules strongly associated with DFUs, yielding 386 candidates. SVM-RFE combined with protein interaction analysis pinpointed four secretory proteins—LYZ, MPO, SLCO2B1 and TREM2—as putative biomarkers. Single-cell analyses showed that MPO, LYZ, SLCO2B1 and TREM2 transcripts are detectable in multiple skin-resident and immune cell populations, and that the DFU-associated upregulation of MPO and LYZ is most pronounced within keratinocyte clusters, while MPO and TREM2 remained consistently dysregulated in independent bulk cohorts. MPO-associated genes were enriched for immune and inflammatory pathways, whereas TREM2-linked genes implicated cell cycle and cytoskeletal regulation. GeneMANIA and ceRNA analyses revealed extensive interaction networks. qRT-PCR confirmed differential expression of MPO and TREM2 in clinical DFU tissues. **Conclusions**: Integrated multi-modal analysis identifies secretory proteins, particularly MPO and TREM2, as central determinants of impaired healing in DFUs. These molecules and their regulatory networks represent promising biomarkers and therapeutic targets for precision management of diabetic wounds.

## 1. Introduction

Diabetic foot ulcers (DFUs) are serious complications of diabetes, affecting up to 25% of patients and responsible for most non-traumatic amputations [1]. They cause poor quality of life and high mortality, with nearly 50% dying within five years [2]. DFUs also generate over $40 billion in annual healthcare costs [1]. Impaired healing in DFUs stems from neuropathy, ischemia, and oxidative stress, leading to chronic inflammation. Elevated cytokines such as TNF-α, IL-1β, and IL-6 disrupt angiogenesis and tissue repair, highlighting the urgent need for better prognostic tools and targeted therapies.

Secretory proteins—including cytokines, chemokines, growth factors, and matrix remodeling enzymes—play central roles in coordinating wound healing. Under normal conditions, healing progresses through overlapping inflammatory, proliferative, and remodeling phases, orchestrated by temporally regulated secretion of these mediators [3]. In the early phase, pro-inflammatory cytokines (e.g., IL-1, IL-6, IL-8, TNF-α) promote immune cell recruitment, while in later phases, anti-inflammatory cytokines and growth factors (e.g., IL-4, IL-10, TGF-β) facilitate angiogenesis and matrix deposition [3]. In diabetes, this regulatory sequence is disrupted, with prolonged inflammatory signaling and insufficient reparative factors [4]. DFUs commonly exhibit excessive inflammation and deficient regenerative signaling, impairing immune cell function, tissue remodeling, and ultimately wound closure [1,4]. This highlights the potential of secretory proteins as key regulators—and biomarkers—of healing outcomes in diabetic wounds.

Despite the clinical demand, reliable molecular biomarkers for predicting DFU healing remain limited. Current evaluations based on ulcer characteristics often fail to predict outcomes accurately [5]. Although various candidates—such as cytokines, proteases, and growth factors—have been explored, their clinical utility is hindered by inconsistent performance across patients. For instance, CXCL6 shows high predictive value for non-healing ulcers, but its systemic inflammatory role reduces specificity [6]. The lack of integrative models and validated biomarker panels further limits clinical translation [5].

Recent advancements in transcriptomics and computational biology offer new tools to decipher DFU pathogenesis. Transcriptome-wide analyses enable the identification of genes and pathways involved in non-healing wounds. Bioinformatics approaches, including gene ontology, protein–protein interactions, and Weighted Gene Co-expression Network Analysis (WGCNA), help isolate wound-related gene modules [7]. Combined with machine learning techniques like least absolute shrinkage and selection operator (LASSO), Support vector machine–recursive feature elimination (SVM-RFE), and random forests, these strategies have identified key regulators such as CXCR4 [7] and mitochondrial targets [8], many of which are secretory or membrane proteins suitable for clinical use.

**Figure 1:**
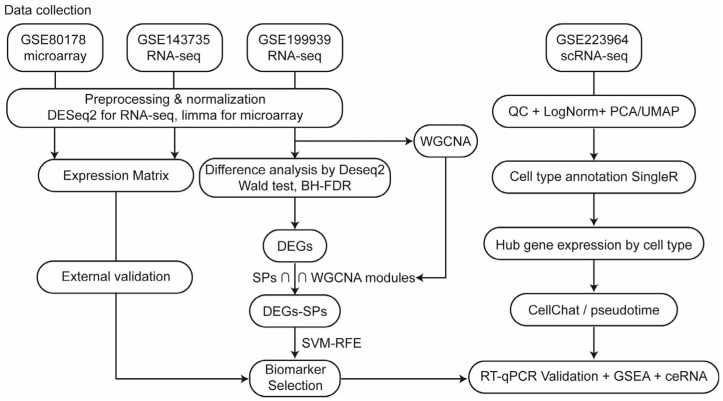
Flowchart of all preprocessing, normalization, filtering, and statistical procedures.

Given the central role of secretory proteins in wound biology and the current gap in reliable DFU biomarkers, we hypothesized that secretory protein-related genes could serve as novel indicators of wound healing status. This study aimed to identify such biomarkers through a combined bioinformatic and experimental approach (Figure 1). By focusing on secretory proteins, we sought to uncover molecules that are both mechanistically involved in impaired wound healing and practically applicable in clinical monitoring. This integrative strategy contributes to a deeper understanding of DFUs’ pathophysiology and offers a potential foundation for precision diagnostics and therapeutic targeting in diabetic wound care.

## 2. Materials and Methods

### 2.1. Data Source and Preprocessing

#### 2.1.1. Data Download

Public gene expression datasets were obtained from the Gene Expression Omnibus (GEO, https://www.ncbi.nlm.nih.gov/geo/, accessed on 10 April 2025). The bulk RNA-seq datasets included GSE199939 (training set), GSE80178, and GSE143735 (validation sets). The training dataset GSE199939 (platform: GPL24676) consisted of 10 diabetic foot ulcer (DFU) and 11 non-DFU foot skin samples. Two independent datasets were used for external validation: GSE80178 (platform: GPL16686), including 9 DFU and 3 normal foot skin samples, and GSE143735 (platform: GPL11154), comprising 9 DFU and 4 non-DFU samples collected from forearm skin. Additionally, the single-cell RNA sequencing dataset GSE223964 (platform: GPL20301) was used to analyze gene expression patterns across different skin cell types, including 5 DFU wounds and 3 non-ulcerated skin samples. A curated list of 3947 secretory protein-coding genes was retrieved from the Human Protein Atlas (https://www.proteinatlas.org/humanproteome/subcellular/secreted+proteins, accessed on 10 April 2025) for the identification of secretory protein-related biomarkers [9]. In this study, GSE199939 was used as the single-platform discovery and training cohort for differential expression, WGCNA and machine-learning-based feature selection, whereas GSE80178 and GSE143735 served only as independent external validation cohorts; expression matrices from different GEO platforms were not merged into a single combined dataset.

#### 2.1.2. Bulk RNA-Seq Data Preprocessing

Three bulk RNA-seq datasets were processed for library-size normalization and gene annotation.

**Figure 2:**
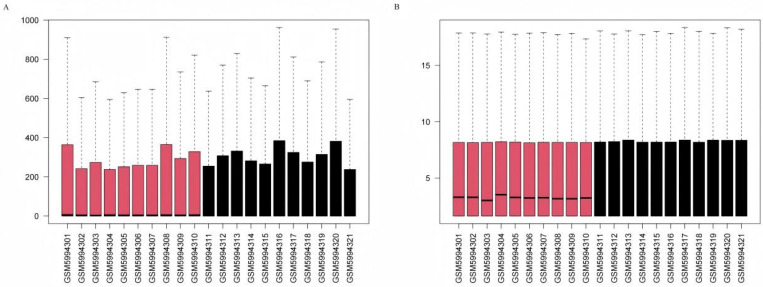
(**A**): Gene expression boxplot before normalization in the GSE199939 dataset. (**B**): Gene expression boxplot after normalization in the GSE199939 dataset. Boxes are colored by phenotype: DFU samples (red, n = 10) and non-DFU control samples (black, n = 11).

GSE199939: The GSE199939 dataset was used as the primary training cohort. As no expression matrix was provided, raw gene-level count data were downloaded from the GEO database and imported into the DESeq2 package (v1.40.2). To account for differences in sequencing depth and RNA composition between samples, sample-wise size factors were estimated using the median-of-ratios method implemented in the estimateSizeFactors function, and raw counts were divided by the corresponding size factor for each sample. The resulting normalized counts were then transformed using the regularized log transformation rlog to stabilize the mean–variance relationship across the dynamic range of expression. Differential expression testing in DESeq2 was performed on the raw count data after size-factor normalization, whereas rlog-transformed values were used only for quality control, clustering, and visualization. After rlog transformation, sample-level quality control was performed by visual inspection of per-sample expression distributions using boxplots and by unsupervised hierarchical clustering to detect potential outliers; no samples showed aberrant behavior and all 21 samples were retained for downstream analyses. Gene identifiers were annotated to official gene symbols based on the GPL24676 platform annotation file. Quality before and after normalization was assessed using boxplots (Figure 2A,B).

GSE80178: For GSE80178, the pre-processed expression matrix was downloaded from GEO and further normalized using the normalizeBetweenArrays function in limma (v3.54.0). Probe IDs were mapped to official gene symbols using the GPL16686 platform annotation. Probes without valid gene symbols were removed, and probes annotated to multiple genes were discarded to avoid ambiguous mapping. When multiple probes corresponded to the same gene symbol, their normalized expression values were averaged to obtain a single gene-level expression value per sample.

GSE143735: Raw count data for GSE143735 were downloaded from GEO and processed using the same DESeq2 (v1.40.2) pipeline as GSE199939: size-factor estimation with estimateSizeFactors to correct for sequencing-depth differences, followed by rlog transformation for variance stabilization. Gene identifiers were annotated using the org.Hs.eg.db package based on Entrez Gene ID mapping and converted to gene symbols accordingly.

#### 2.1.3. Single-Cell RNA-Seq Data Preprocessing

The single-cell RNA sequencing dataset GSE223964 was obtained from the GEO database. All preprocessing and downstream analyses were performed using the Seurat R package (v4.1.1).

**Figure 3:**
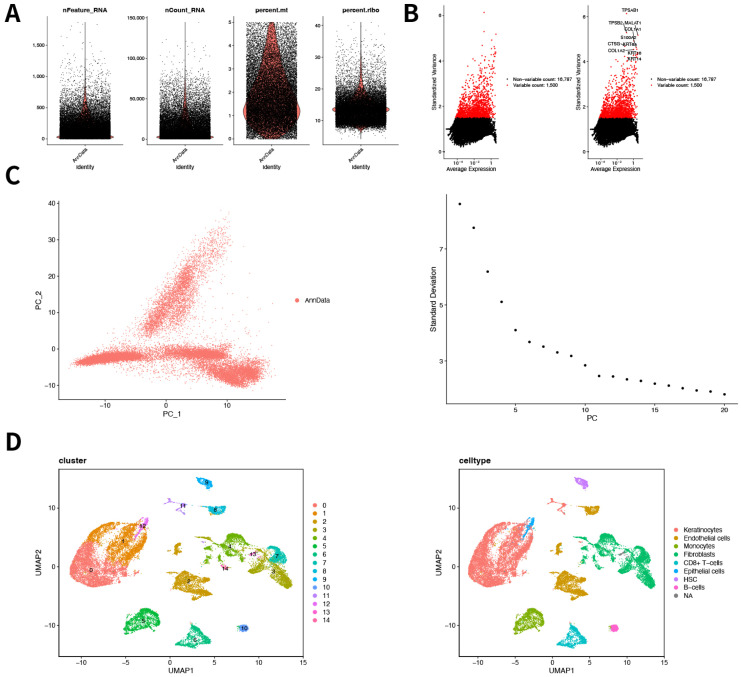
Single-cell RNA Sequencing Data Analysis. (**A**): Distribution of quality control metrics, including nFeature_RNA, nCount_RNA, percent.mt, and percent.ribo. Red color indicates the DFU group, and black color indicates the non-DFU control group. (**B**): Scatter plots of standardized variance versus average expression, highlighting highly variable genes. (**C**): Principal Component Analysis (PCA). The left plot shows cell distribution based on the first two principal components (PC1 and PC2). The right plot depicts the standard deviation across the principal components. (**D**): Uniform Manifold Approximation and Projection (UMAP) analysis. The left plot displays identified cell clusters, while the right plot provides annotations of different cell types.

Quality Control: Initial quality filtering was applied using the Seurat package with the following criteria: cells with fewer than 50 detected features, genes expressed in fewer than 3 cells, or cells with >5% mitochondrial gene content were excluded. Cell retention after filtering is illustrated in Figure 3A.

Normalization and Feature Selection: Normalization was conducted using the LogNormalize method. Highly variable genes were identified using the FindVariableFeatures function, which calculates gene variability based on the mean–variance relationship. The top 1500 highly variable genes were selected for downstream analysis. The most variable top 10 genes were highlighted using the LabelPoints function and visualized in Figure 3B.

Scaling and Dimensionality Reduction: Data scaling was performed using the ScaleData function. Principal component analysis (PCA) was conducted on the top 1500 variable genes using the RunPCA function. The JackStrawPlot function was used to evaluate the statistical significance of each principal component. The top 20 statistically significant principal components (*p* < 0.05) were selected for further dimensionality reduction, as shown in Figure 3C. Principal component analysis (PCA) was therefore applied only within the GSE223964 single-cell dataset and was not used to jointly embed bulk samples from different platforms.

Clustering and Cell Type Annotation: Unsupervised clustering was performed using the FindNeighbors and FindClusters functions, based on the top 10 PCA components. A clustering resolution of 0.2 was applied. Cluster visualization was carried out using Uniform Manifold Approximation and Projection (UMAP), shown in Figure 3D. Cell type annotation was conducted using the SingleR package v1.12.0 in conjunction with the CellMarker database, matching clusters to known cell types. Annotated clusters were visualized using UMAP (Figure 3D).

### 2.2. Differential Expression and Secretory Protein Intersection Analysis

To identify differentially expressed genes (DEGs) between DFU and control samples in the training dataset, the “DESeq2” package (v1.40.2) was used for differential expression analysis. Genes with an absolute log2 fold change (|log2FC|) > 1 and an adjusted *p*-value < 0.05 were considered statistically significant DEGs. The volcano plot of DEGs was generated using the “ggplot2” package (v3.4.1), and the expression patterns of the top 50 DEGs ranked by adjusted *p*-value were visualized using the “pheatmap” package (v1.0.12). To identify secretory protein-related DEGs, the resulting DEG list was intersected with a reference list of 3947 secretory protein-coding genes curated from the Human Protein Atlas secretome database. The overlap between these gene sets was visualized using the “VennDiagram” package (v1.7.3). All differential expression testing in this study was carried out in the GSE199939 training cohort, and samples from GSE80178 and GSE143735 were not included in DEG model fitting.

### 2.3. Weighted Gene Co-Expression Network Analysis

**Figure 4:**
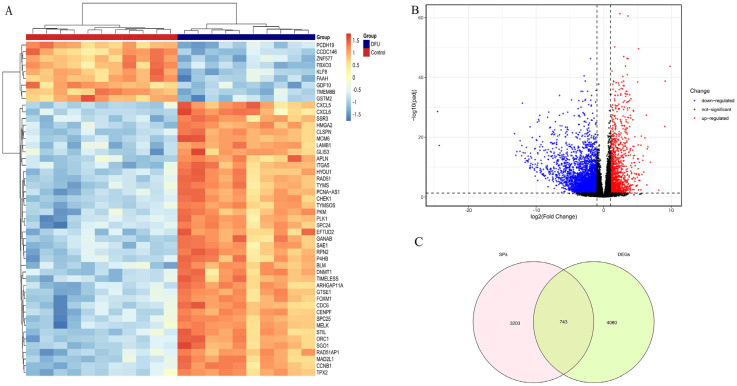
Identification of Differentially Expressed Genes (DEGs) and secretory proteins (SPs) in Diabetic Foot Ulcers (DFUs). (**A**): Heatmap illustrating the hierarchical clustering of differentially expressed genes between DFU samples and control groups. (**B**): Volcano plot showing significantly upregulated (red), downregulated (blue), and non-significant (black) genes based on fold change and adjusted *p*-value thresholds. (**C**): Venn diagram demonstrating the overlap between identified secretory proteins (SPs) and differentially expressed genes (DEGs).

WGCNA was performed using the” WGCNA” package to identify gene modules associated with DFU. Before network construction, unsupervised hierarchical clustering of all 21 samples was conducted to identify potential outliers; as no outlier samples were observed in the sample dendrogram (Figure 4A), all samples were included in the WGCNA. A soft-thresholding power was selected to achieve scale-free topology. Gene modules were constructed with a minimum module size of 30, and modules were merged using a height cutoff of 0.25. Modules significantly associated with DFU were selected for downstream analysis. The genes within these modules were intersected with secretory protein-related DEGs to obtain candidate genes for further evaluation [10,11]. The co-expression network was constructed using normalized expression data from GSE199939 only, and expression matrices from GSE80178 and GSE143735 were not used in the WGCNA step.

### 2.4. Machine Learning-Based Feature Selection

SVM-RFE was applied to identify optimal diagnostic biomarkers from the set of candidate genes obtained from WGCNA. The analysis was implemented using the “e1071” R package (v1.7-16), and model performance was evaluated through five-fold cross-validation. SVM-RFE feature selection and model training were performed exclusively in the GSE199939 training cohort; the GSE80178 and GSE143735 datasets were reserved as independent test sets and were not used for model training or cross-validation.

### 2.5. Functional Enrichment

To understand the biological pathways in which the candidate genes might be involved, Gene Ontology (GO) and Kyoto Encyclopedia of Genes and Genomes (KEGG) pathway enrichment analysis for the candidate genes was conducted using “clusterProfiler” (v4.2.2) with an adjusted *p*-value < 0.05. The GO categories included biological processes (BP), cellular components (CC), and molecular functions (MF).

### 2.6. Protein–Protein Interaction (PPI) Network Construction and Hub Gene Analysis

To identify key secretory protein-related genes in diabetic foot ulcers (DFU), the candidate genes selected through machine learning algorithms were imported into the STRING database (https://cn.string-db.org/, accessed on 10 April 2025) to construct a protein–protein interaction (PPI) network. Interacting genes with either direct physical associations or functionally predicted relationships were extracted from the network and defined as hub genes. The PPI network was generated and visualized directly using the STRING web interface.

To further explore the relationships among these hub genes, Spearman correlation analysis was performed using the “psych” R package (v2.1.6) based on gene expression profiles from the GSE199939 training dataset. Correlation coefficients were calculated to assess the strength of pairwise associations among hub genes and visualized in a correlation matrix.

In addition, the differential expression of hub genes between DFU and control samples was assessed using boxplots generated by the “ggplot2” R package (v3.4.1). Statistical comparisons were conducted using the Wilcoxon rank-sum test, and *p*-values less than 0.05 were considered statistically significant.

### 2.7. Single-Cell RNA-Seq Analysis of Hub Genes

#### 2.7.1. Expression of Hub Genes in Single-Cell Subpopulations

To characterize the distribution of hub gene expression across different cell types, the normalized scRNA-seq dataset (GSE223964) was analyzed using the Seurat package (v4.1.1). The expression profiles of each hub gene were visualized by cell type using feature plots and dot plots. Mean expression values across cell types were also computed to assess relative abundance [12].

To compare gene expression levels between diabetic foot ulcer (DFU) and control groups within each annotated cell type, differential expression analysis was performed. Boxplots were generated using the ggplot2 package (v3.4.1) to illustrate the expression distribution of hub genes in each cell type under DFU and control conditions. The Wilcoxon rank-sum test was used to determine statistical significance (*p* < 0.05).

#### 2.7.2. Cell–Cell Communication Analysis

To infer intercellular communication between annotated cell types, the CellChat package (v1.1.3) was applied to the scRNA-seq dataset. Ligand–receptor interactions were predicted based on the CellChatDB.human reference database. The analysis calculated the probability and strength of communication between cell pairs, enabling visualization of both global cell–cell interaction networks and specific pathways involving hub gene-expressing cell types.

To evaluate differences in cell communication between DFU and control groups, interaction probability and statistical significance (*p*-values) were calculated. Scatter plots were generated using ggplot2 to compare the communication activity of each cell pair across conditions [13].

#### 2.7.3. Pseudotime Trajectory Analysis

To explore the dynamic expression of hub genes during cellular transitions, pseudotime trajectory analysis was conducted on hub gene-expressing cells. Subclustering was first performed using Seurat’s FindNeighbors and FindClusters functions (resolution = 0.2), enabling detection of fine-grained cellular subpopulations. Dimensionality reduction and visualization were carried out using UMAP.

Cells enriched for hub gene expression were subjected to trajectory inference using the Monocle2 package (v2.22.0). Pseudotemporal ordering was reconstructed to infer cellular lineage progression. The differentialGeneTest function was used to identify genes dynamically expressed along the pseudotime axis. Hub genes were specifically evaluated for their consistency with differentiation trends.

Trajectory branches were segmented according to inferred developmental nodes, and the expression patterns of prognostic markers were compared across pseudotime-defined stages. This analysis was independently performed for each relevant cell type to evaluate their role in DFU-related pathological remodeling.

### 2.8. External Validation and Diagnostic Evaluation

The expression of candidate biomarkers was validated in the GSE80178 and GSE143735 datasets. Receiver operating characteristic (ROC) curve analysis was performed using the “pROC” package (v1.18.0) to assess diagnostic performance. Genes with an area under the curve (AUC) > 0.70 were considered robust biomarkers. For both GSE80178 and GSE143735, normalization, ROC curve construction and AUC estimation were performed within each cohort independently, without pooling samples into a cross-platform expression matrix.

Group-wise expression differences were evaluated using the Wilcoxon rank-sum test, implemented in base R (v4.2.2). Boxplots were generated using the “ggplot2” package (v3.4.1) to visualize differential expression between DFU and control samples. Group-wise comparisons were performed separately within each dataset to avoid cross-platform confounding.

### 2.9. Functional Enrichment and Regulatory Network Analysis

To explore the biological functions and regulatory associations of key candidate genes, multiple downstream analyses were performed.

Single-gene Gene Set Enrichment Analysis (GSEA) was conducted for individual biomarkers based on Kyoto Encyclopedia of Genes and Genomes (KEGG) pathway gene sets. Genes were ranked according to Spearman correlation coefficients with each biomarker. GSEA was implemented using the “clusterProfiler” package (v4.2.2) in R (v4.2.2) [14].

To investigate gene-level functional interactions, gene co-expression networks were constructed using GeneMANIA (accessed via https://genemania.org/ on 10 April 2025, Cytoscape plugin version 3.5.2). Candidate genes were used as input nodes to retrieve functionally associated genes based on co-expression, physical interaction, shared pathway, and co-localization evidence.

Additionally, a predicted competing endogenous RNA (ceRNA) networks involving long noncoding RNAs (lncRNAs), microRNAs (miRNAs), and target mRNAs were inferred in silico using the RNAInter database (http://www.rnainter.org/; interaction score threshold > 0.7). All interaction networks were visualized and edited using Cytoscape software (v3.8.2) [15]. These interactions are computational predictions and have not yet been experimentally validated.

### 2.10. qRT-PCR Experiment

To experimentally validate whether the expression levels of the identified biomarkers in diabetic foot ulcer (DFU) tissues are consistent with bioinformatics predictions, skin tissue samples were collected from 15 DFU patients at Capital Medical University Affiliated Beijing Shijitan Hospital. For each patient, both ulcer tissue and adjacent non-ulcerated (normal) skin tissue from the same individual were obtained. All participants provided written informed consent prior to sample collection. The study protocol was reviewed and approved by the Ethics Committee of Capital Medical University Affiliated Beijing Shijitan Hospital (Approval No. IIT2025-002-002). Total RNA was isolated using TRIzol reagent (Invitrogen, 15596018, Carlsbad, CA, USA) according to the manufacturer’s instructions. First-strand complementary DNA (cDNA) was synthesized using the Hifair^®^ III 1st Strand cDNA Synthesis SuperMix for qRT-PCR kit (Yeasen Biotechnology, 11141ES60, Shanghai, China). All gene-specific primers were synthesized by Shanghai Shengong Biotech Co., Ltd. real-time quantitative reverse transcription PCR (qRT-PCR) was performed in three independent biological replicates for each sample. GAPDH was used as the internal reference gene, and relative expression levels of the target biomarkers were calculated using the 2^−ΔΔCt^ method. Differences in biomarker expression between DFU and control samples were statistically analyzed using a two-tailed Student’s *t*-test in GraphPad Prism 10 software. A *p*-value of <0.05 was considered statistically significant. Details of the qRT-PCR reaction system, thermocycling conditions, and specific primer sequences used are provided in the Supplementary Tables S1–S5.

### 2.11. Statistical Analysis

All statistical analyses were performed using R statistical software (v 4.2.2). Differences between two groups were compared using the Wilcoxon rank-sum test (*p* < 0.05). In RT-qPCR analysis, statistical comparisons were made using the *t*-test (*p* < 0.05). In violin plots, box plots, and heatmaps, **** indicated *p* < 0.0001, *** indicated *p* < 0.001, ** indicated *p* < 0.01, * indicated *p* < 0.05, and ns indicated *p* > 0.05.

## 3. Results

### 3.1. Identification of Differentially Expressed Secretory Protein Genes

A total of 4803 differentially expressed genes (DEGs) were identified between DFU and non-DFU samples in the training dataset GSE199939, including 1284 upregulated and 3519 downregulated genes (Figure 4A,B). Intersecting these DEGs with 3947 curated secretory protein-coding genes yielded 743 differentially expressed secretory protein-related genes (DEGs-SPs) (Figure 4C), representing a refined set of secretory targets relevant to DFU pathology.

### 3.2. Co-Expression Network Construction and Candidate Gene Selection

To explore the functional co-expression patterns of differentially expressed secretory protein-related genes, WGCNA was conducted using the R package, v4.1.1, WGCNA. The input matrix included DEGs from the training dataset GSE199939, and the phenotype trait was defined as DFU vs. control status.

**Figure 5:**
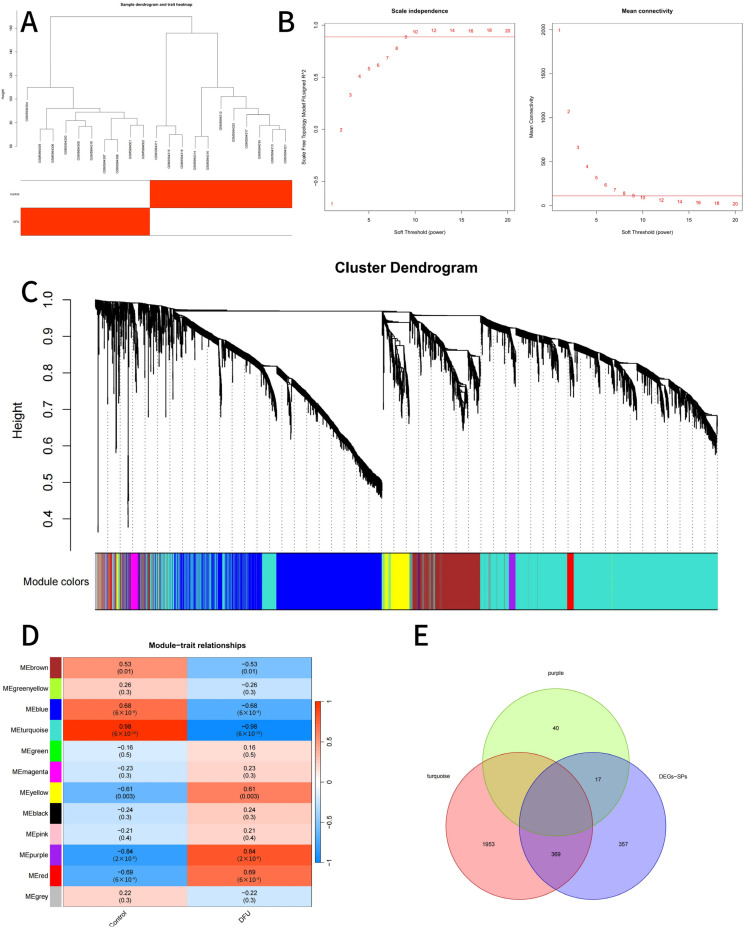
WGCNA and Module-Trait Relationship Identification. (**A**): Sample dendrogram and trait heatmap illustrating sample clustering and association with clinical traits. The red color highlights samples belonging to the DFU group in the trait heatmap. (**B**): Determination of the soft-thresholding power. Left: Scale independence analysis; Right: Mean connectivity for various soft-thresholding powers. (**C**): Cluster dendrogram of genes identified through hierarchical clustering. Colored bars represent distinct gene modules. (**D**): Heatmap showing correlations between identified gene modules and clinical traits (Control and DFU). Red indicates positive correlation, blue indicates negative correlation, and the intensity represents the strength of correlation. (**E**): Venn diagram depicting the intersection among critical modules (purple and turquoise) and the combined list of differentially expressed genes and secretory proteins (DEGs + SPs).

Sample clustering analysis showed no outliers among the 21 samples; thus, no samples were removed in the initial quality control step (Figure 5A). To ensure scale-free topology in the network, the pickSoftThreshold function was used to determine the optimal soft-thresholding power. A power value of 9 was selected, as it satisfied the degree of independence threshold (>0.887) (Figure 5B).

Network construction was then performed with the selected power = 9. The minimum module size (minModuleSize) was set to 30, and modules were merged with a mergeCutHeight of 0.25. A total of 12 distinct gene modules were identified, each labeled with a unique color (Figure 5C).

The correlation between module eigengenes and DFU phenotype was then assessed. Among the 12 modules, the purple module (cor = −0.98, *p* = 2 × 10^−15^) and the turquoise module (cor = 0.84, *p* = 2 × 10^−6^) showed the strongest associations with DFU status (Figure 5D). These two key modules contained 57 genes (purple) and 2322 genes (turquoise), respectively.

To further narrow down candidate genes, an intersection was performed between the genes in the two DFU-related modules and the previously identified 743 differentially expressed secretory protein-related genes (DEGs-SPs). This yielded a refined list of 386 candidate genes, as visualized using a Venn diagram (Figure 5E).

### 3.3. SVM-RFE Feature Selection and Biomarker Identification

**Figure 6:**
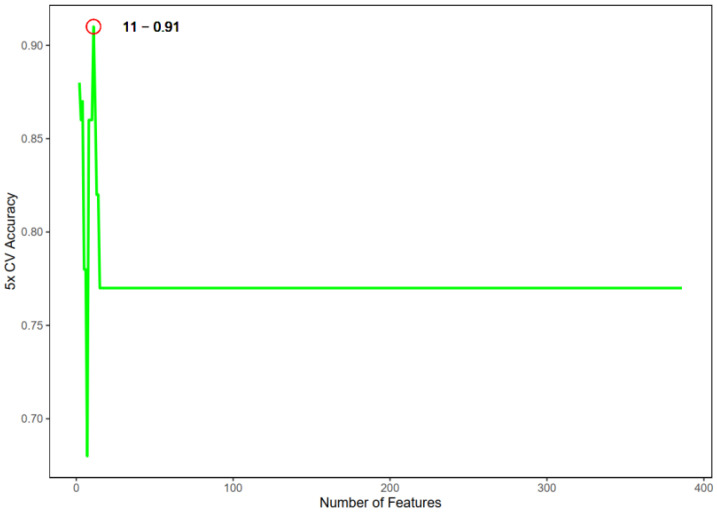
Relationship between the number of SVM-selected features and model accuracy. The green line represents the 5-fold cross-validated accuracy across increasing numbers of features, while the red circle highlights the optimal feature number (11 features) corresponding to the highest accuracy (0.91).

To refine diagnostic biomarkers, SVM-RFE was applied to the 386 candidate genes. Cross-validation identified the optimal feature number as 11, yielding a maximum classification accuracy of 0.91 (Figure 6). The selected genes included FAM78A, LGMN, TAFA5, LYZ, DRAM1, C2, TREM2, MPO, PTPRO, SLCO2B1, and OTOA.

### 3.4. Functional Enrichment Analysis of Biomarkers

**Figure 7:**
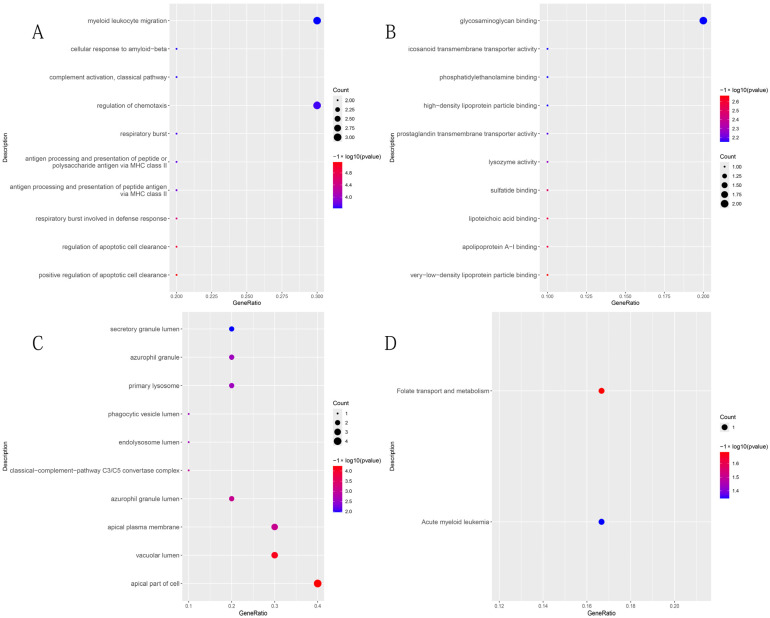
Functional Enrichment Analysis of Identified Key Genes. (**A**): Gene Ontology (GO) enrichment analysis for biological processes (BP), highlighting significantly enriched processes such as myeloid leukocyte migration and chemotaxis. (**B**): GO enrichment analysis for molecular functions (MF), identifying functions such as glycosaminoglycan binding and transporter activities. (**C**): GO enrichment analysis for cellular components (CC), emphasizing the involvement of secretory granule lumen, azurophil granules, and lysosomal components. (**D**): Kyoto Encyclopedia of Genes and Genomes (KEGG) pathway analysis, illustrating significantly enriched pathways including folate transport and metabolism and acute myeloid leukemia pathways. The size of each dot represents the count of genes involved, and the color intensity indicates the significance level (–log10(*p*-value)).

Gene Ontology (GO) and KEGG pathway enrichment analyses revealed that the 11 candidate genes were involved in 433 biological processes (BP) (Figure 7A), 33 molecular functions (MF) (Figure 7B), and 21 cellular components (CC) (Figure 7C), as well as 2 significantly enriched KEGG pathways (Figure 7D), indicating a strong association with immune regulation and inflammation.

### 3.5. PPI Network and Hub Gene Identification

**Figure 8:**
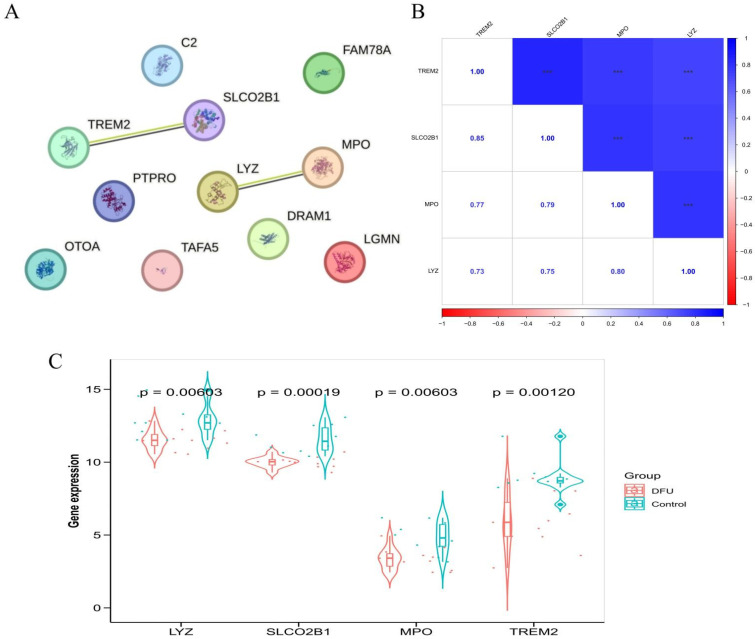
Protein Interaction Network, Correlation Analysis, and Expression Validation of Key Genes. (**A**): Protein–protein interaction (PPI) network depicting interactions among key proteins identified from the analysis. (**B**): Correlation heatmap illustrating pairwise correlation coefficients among the selected key genes (TREM2, SLCO2B1, MPO, LYZ). Blue indicates positive correlations, while red indicates negative correlations. Color intensity represents correlation strength. (**C**): Violin plots demonstrating differential expression levels of key genes (LYZ, SLCO2B1, MPO, TREM2) between diabetic foot ulcer (DFU) samples and control groups. Statistical significance (*p*-values) is indicated for each comparison. *** indicates statistical significance (*p* < 0.001).

Protein–protein interaction (PPI) analysis using the STRING database identified 4 hub genes (LYZ, MPO, SLCO2B1, and TREM2) with direct or functionally predicted interactions among the 11 candidates (Figure 8A). Spearman correlation analysis showed significant co-expression among these hub genes in the GSE199939 dataset (Figure 8B). All four hub genes were differentially expressed between DFU and control samples (*p* < 0.05), as visualized by boxplots (Figure 8C).

### 3.6. Single-Cell Expression, Intercellular Communication, and Differentiation Dynamics of Hub Genes

#### 3.6.1. Single-Cell Expression and Cellular Distribution of Hub Genes

**Figure 9:**
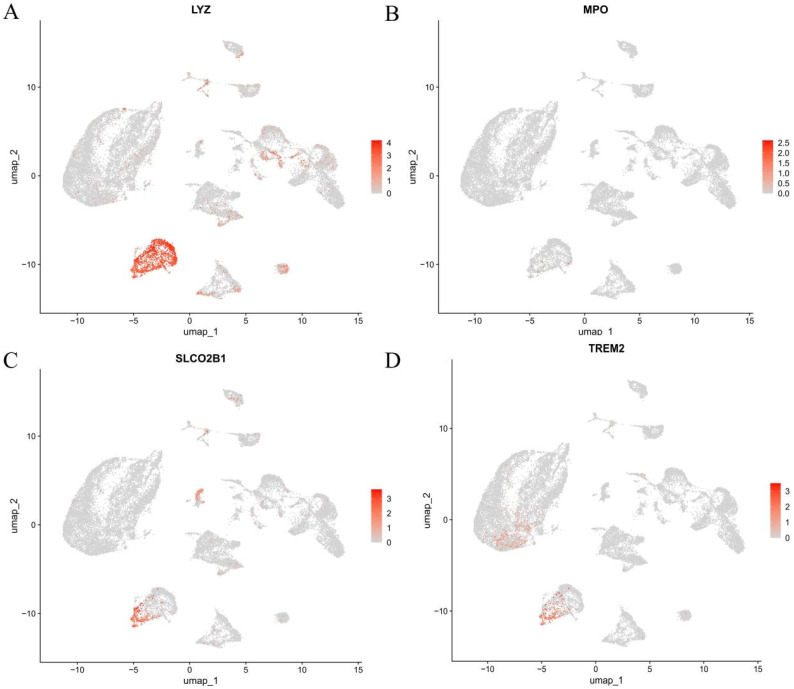
Single-cell Expression Patterns of Key Genes in Diabetic Foot Ulcer Tissues. UMAP plots visualizing the expression levels of key genes within single-cell populations: (**A**): Expression distribution of LYZ. (**B**): Expression distribution of MPO. (**C**): Expression distribution of SLCO2B1. (**D**): Expression distribution of TREM2. Color intensity represents the level of gene expression, with darker red indicating higher expression.

**Figure 10:**
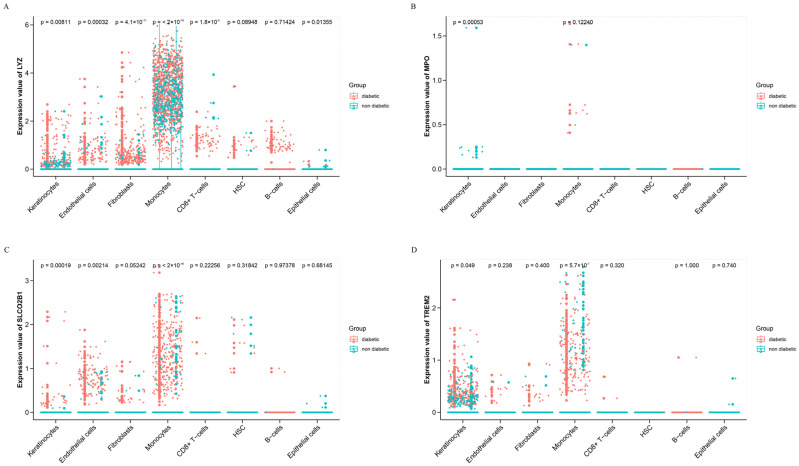
Comparative Analysis of Key Gene Expression Across Cell Types in Diabetic and Non-Diabetic Groups. Expression comparison of key genes across different cell populations between diabetic and non-diabetic groups: (**A**): LYZ gene expression. (**B**): MPO gene expression. (**C**): SLCO2B1 gene expression. (**D**): TREM2 gene expression. Each plot indicates gene expression levels in distinct cell types. Statistical significance (*p*-values) is provided for each comparison. Red points represent diabetic samples, and blue points represent non-diabetic controls.

To investigate the cellular localization and regulatory expression patterns of key biomarkers at single-cell resolution, we analyzed the scRNA-seq dataset GSE223964. Four hub genes—LYZ, MPO, SLCO2B1, and TREM2—were projected onto Uniform Manifold Approximation and Projection (UMAP) plots, revealing distinct cell-type-specific expression patterns. To investigate the cellular localization of the hub genes, we projected LYZ, MPO, SLCO2B1 and TREM2 onto the UMAP embedding of the GSE223964 single-cell dataset. The feature plots showed that transcripts of all four genes were detectable in several cell populations, including keratinocytes, fibroblasts, endothelial cells and myeloid cells, rather than being restricted to a single lineage (Figure 9A–D). Thus, Figure 8 is used to map the spatial distribution of these genes across the single-cell landscape, not to redefine MPO and LYZ as keratinocyte-specific markers. In subsequent analyses (Figure 10), we compared gene expression between DFU and non-ulcer samples within each annotated cell type and found that the difference in MPO and LYZ expression between DFU and control was most pronounced in keratinocyte clusters, whereas changes in other cell types were less marked. We therefore focused on keratinocytes as the compartment in which DFU-associated dysregulation of these genes was most evident, while acknowledging that MPO and LYZ remain classical neutrophil/monocyte markers and that their signal in keratinocytes may partly reflect inflammatory crosstalk in the ulcer microenvironment.

In line with the feature and dot plots, the cell-type–resolved boxplots further demonstrate that DFU-associated dysregulation of the four hub genes is most coherent within keratinocytes. In keratinocyte clusters, DFU samples showed significantly higher expression of LYZ (*p* = 0.0081), MPO (*p* = 0.00053), SLCO2B1 (*p* = 0.00019) and TREM2 (*p* = 0.049) compared with non-ulcerated skin, indicating a coordinated shift of this gene set in the epidermal compartment. At the same time, LYZ and SLCO2B1 were also markedly upregulated in fibroblasts, endothelial cells and monocytes (for example, LYZ and SLCO2B1 in fibroblasts and monocytes, *p* values < 10^−11^), whereas changes in B cells and epithelial cells were minimal or not statistically significant. These patterns support the view that MPO and LYZ remain broadly expressed neutrophil/monocyte-associated genes at the tissue level, while the difference between DFU and control is particularly evident in keratinocytes, rather than implying that these genes are exclusively or maximally expressed in keratinocytes (Figure 10A–D).

#### 3.6.2. Intercellular Communication Alterations in the DFU Microenvironment

To elucidate the dynamics of cell–cell communication in diabetic foot ulcers (DFUs), intercellular signaling analysis was performed using the CellChat framework based on the scRNA-seq dataset GSE223964. A total of eight major cell types were analyzed, including keratinocytes, endothelial cells, fibroblasts, monocytes, CD8^+^ T cells, hematopoietic stem cells (HSCs), B cells, and epithelial cells.

**Figure 11:**
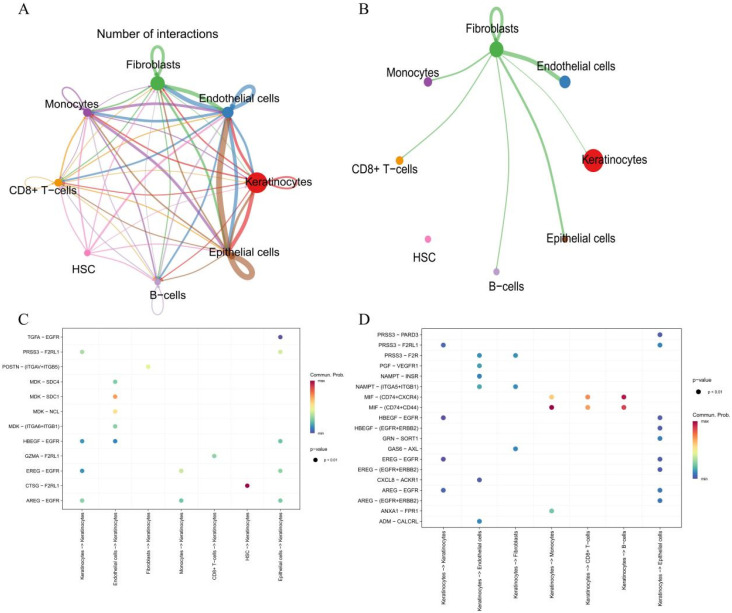
Cell Communication Network and Signaling Interactions in Diabetic Foot Ulcer Microenvironment. (**A**): Overview of cell–cell communication network. Lines indicate interactions, and line thickness corresponds to the number of interactions between cell types. (**B**): Signaling interactions initiated by fibroblasts with other cell populations, highlighting fibroblasts as key signaling initiators. (**C**): Bubble plot depicting significant ligand-receptor interactions originating from various cell types targeting keratinocytes. (**D**): Bubble plot illustrating significant ligand-receptor interactions from keratinocytes targeting other cell types. Bubble color indicates communication probability, and bubble size represents the statistical significance (*p*-value). Each colored line corresponds to the signaling interactions originating from a specific cell type, with colors matching the nodes: keratinocytes (red), endothelial cells (dark blue), fibroblasts (green), monocytes (purple), CD8^+^ T cells (orange), HSC (pink), B cells (light blue), and epithelial cells (brown). Thicker lines indicate stronger or more abundant ligand–receptor interactions.

Quantitative analysis of interaction networks revealed a marked increase in the total number and strength of interactions in DFU samples compared to controls (Figure 11A). Notably, keratinocytes exhibited the most substantial expansion in interaction quantity and signal weight, functioning as both dominant information senders and receivers (Figure 11B).

Dot plot analysis of ligand–receptor interactions directed toward keratinocytes indicated significantly upregulated signaling pathways in DFU samples. These included growth factor–mediated interactions such as HBEGF–EGFR, AREG–EGFR, and TGFA–EGFR, as well as protease-activated receptor interactions like PRSS3–F2RL1 and CTSG–F2RL1, suggesting a shift toward an inflammatory and proliferative signaling state (Figure 11C).

Conversely, when keratinocytes were analyzed as signaling sources, enhanced outward communication was observed toward endothelial cells, fibroblasts, immune cells, and epithelial populations. Key outgoing ligand–receptor pairs such as CXCL8–ACKR1, GAS6–AXL, GRN–SORT1, and MIF–(CD74 + CXCR4/CD44) were significantly enriched, indicating that keratinocytes actively modulate angiogenesis, immune recruitment, and matrix remodeling in DFU tissue (Figure 11D). Taken together, these findings suggest that keratinocytes play a central regulatory role in the altered cell–cell communication network of DFUs, participating in both autocrine and paracrine signaling circuits that may contribute to chronic inflammation and impaired wound healing.

#### 3.6.3. Pseudotime Trajectory Analysis of Hub Genes in Keratinocytes

To further investigate the dynamic regulatory role of hub genes in keratinocyte state transitions during diabetic wound healing, pseudotime trajectory analysis was performed using Monocle2 based on scRNA-seq data from the GSE223964 dataset.

**Figure 12:**
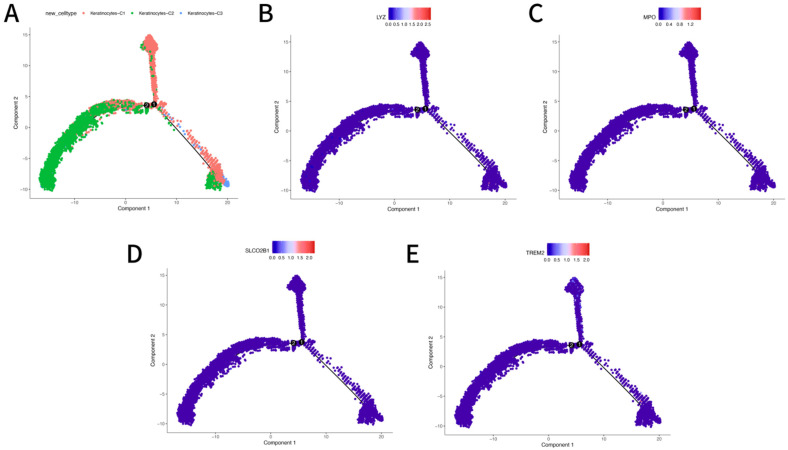
Pseudotime Trajectory Analysis of keratinocyte Subpopulations and Expression Patterns of Key Genes. (**A**): Pseudotime trajectory analysis visualizing distinct keratinocyte subpopulations (Keratinocyte–C1, Keratinocyte–C2, Keratinocyte–C3) along developmental states. (**B**–**E**): Expression patterns of key genes across the pseudotime trajectory: (**B**): LYZ gene expression. (**C**): MPO gene expression. (**D**): SLCO2B1 gene expression. (**E**): TREM2 gene expression. Color intensity (blue to red gradient) indicates gene expression levels, with red denoting higher expression.

The reconstructed cell lineage trajectory identified multiple differentiation branches within the keratinocyte population, suggesting a complex cellular response landscape in DFU. Keratinocytes from DFU samples exhibited a shifted and expanded pseudotime distribution, indicating altered cell fate progression compared to controls (Figure 12A).

Projection of individual hub gene expression onto the pseudotime trajectory revealed temporal heterogeneity in expression patterns. LYZ expression increased progressively along pseudotime, peaking in terminal differentiation states, suggesting its role in late-stage keratinocyte activation or inflammatory response (Figure 12B). MPO showed moderate expression with a biphasic pattern, suggesting dynamic regulation in transitional cellular states (Figure 12C). SLCO2B1 displayed a rising trend early in the pseudotemporal axis, indicating possible involvement in early-phase metabolic or signaling adaptation (Figure 12D). TREM2 demonstrated an overall low but detectable expression, peaking mid-trajectory, which may relate to immunomodulatory signaling during keratinocyte plasticity (Figure 12E). Together, these pseudotime analyses suggest that the four hub genes are dynamically regulated throughout keratinocyte differentiation, and may participate in modulating wound repair, inflammation, and epidermal remodeling in DFU.

### 3.7. External Validation of Biomarkers in Independent Cohorts

**Figure 13:**
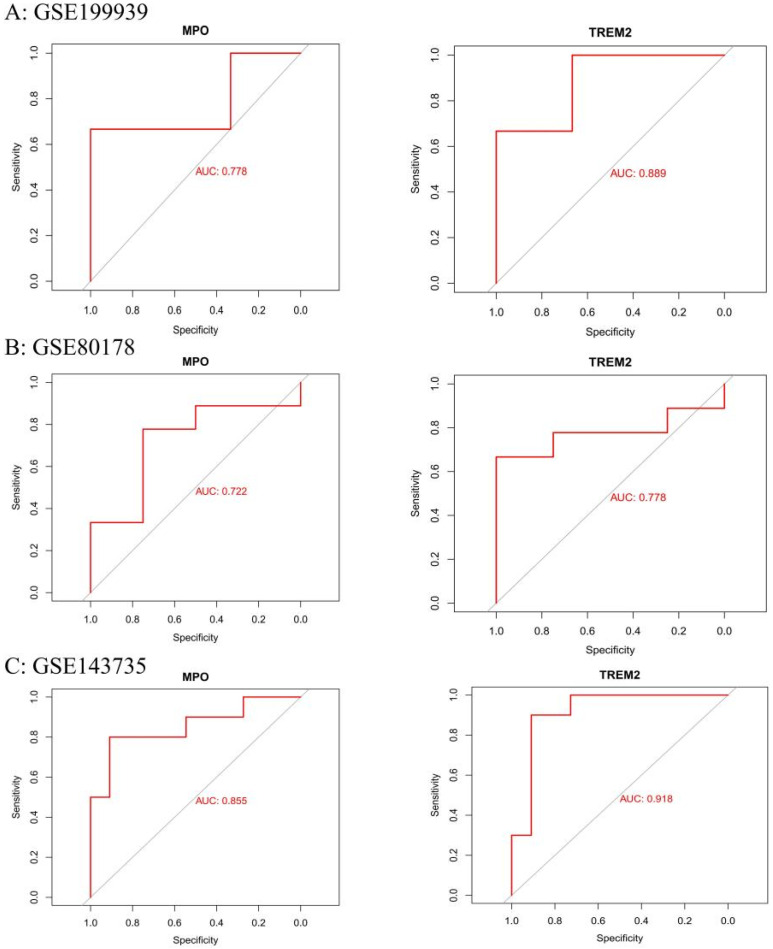
Receiver Operating Characteristic (ROC) Curve Analysis for Diagnostic Efficacy of Key Genes. ROC curves evaluating the diagnostic potential of key genes (MPO, TREM2) in diabetic foot ulcers (DFU) across three independent datasets: (**A**): ROC curves based on dataset GSE199939. (**B**): ROC curves based on dataset GSE80178. (**C**): ROC curves based on dataset GSE143735. The area under the curve (AUC) values, indicated in each plot, reflect the diagnostic accuracy of each gene. A higher AUC value represents better diagnostic performance.

Based on ROC curve analysis across both the training (GSE199939) and validation datasets (GSE80178 and GSE143735), only MPO and TREM2 consistently demonstrated diagnostic value, with AUC values exceeding 0.70, indicating their potential as reliable biomarkers for distinguishing DFU from non-ulcerated tissues (Figure 13A–C).

**Figure 14:**
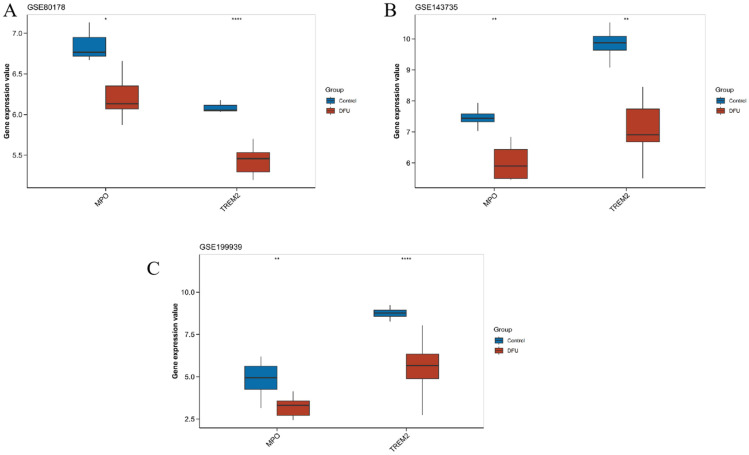
Validation of Key Gene Expression in Independent Diabetic Foot Ulcer Datasets. Comparative box plots validating the expression levels of key genes (MPO, TREM2) in diabetic foot ulcer (DFU) samples versus control samples across independent datasets: (**A**): Validation results from dataset GSE80178. (**B**): Validation results from dataset GSE143735. (**C**): Validation results from dataset GSE199939. Asterisks indicate statistical significance levels (* *p* < 0.05; ** *p* < 0.01; **** *p* < 0.0001). DFU samples are represented in red, and control samples in blue.

Furthermore, expression analysis using Wilcoxon tests confirmed that both genes were significantly downregulated in DFU samples across all cohorts (*p* < 0.05), with consistent directional trends (Figure 14A–C). These results support the robustness and reproducibility of MPO and TREM2 as secretory protein-related biomarkers with clinical relevance for DFU diagnosis and pathophysiological monitoring.

### 3.8. Functional Annotation of Hub Genes

#### 3.8.1. Functional Characterization of Hub Genes via Single-Gene GSEA

**Figure 15:**
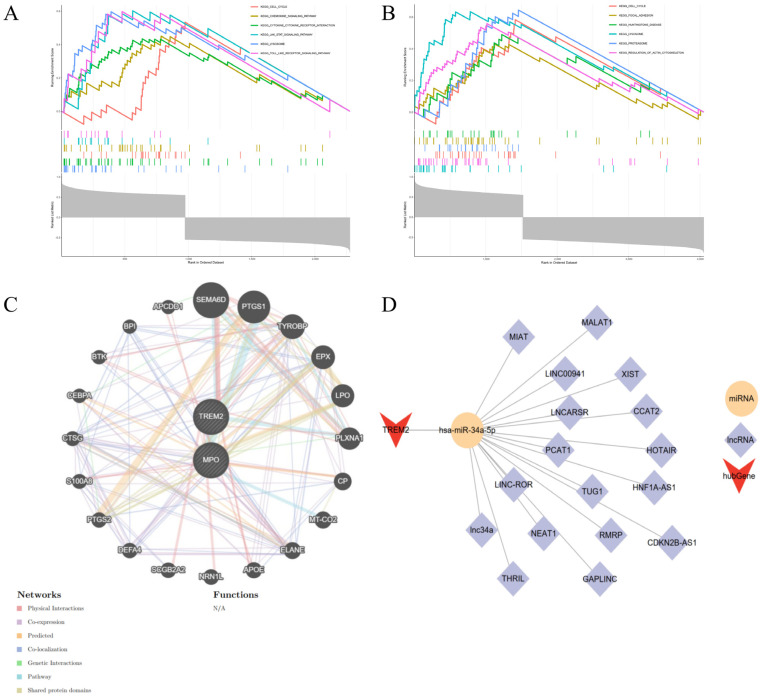
Gene Set Enrichment Analysis (GSEA) and Interaction Networks of Key Genes in Diabetic Foot Ulcers. (**A**,**B**): Gene Set Enrichment Analysis (GSEA) illustrating significant enrichment of gene sets associated with key genes, highlighting pathways potentially involved in diabetic foot ulcer pathology. (**C**): Protein interaction network depicting multiple interaction types (e.g., physical interaction, co-expression, predicted interaction) among proteins related to key genes. (**D**): Competitive endogenous RNA (ceRNA) network showing interactions involving miRNA (hsa-miR-34a-5p), hub gene (TREM2), and related long non-coding RNAs (lncRNAs).

For MPO, GSEA revealed significant positive enrichment in multiple immune- and inflammation-related pathways, including KEGG_CHEMOKINE_SIGNALING_PATHWAY, KEGG_TOLL_LIKE_RECEPTOR_SIGNALING_PATHWAY, and KEGG_CYTOKINE_CYTOKINE_RECEPTOR_INTERACTION. Additional enriched pathways included KEGG_LYSOSOME, KEGG_JAK_STAT_SIGNALING_PATHWAY, and KEGG_CELL_CYCLE (Figure 15A). These results are consistent with MPO’s known role in neutrophil activity, oxidative stress, and immune regulation in chronic wounds.

For TREM2, the top six KEGG pathways enriched among genes positively correlated with its expression included KEGG_REGULATION_OF_ACTIN_CYTOSKELETON, KEGG_FOCAL_ADHESION, and KEGG_CELL_CYCLE, indicating involvement in cytoskeletal remodeling and cell proliferation. Additionally, TREM2 was associated with KEGG_LYSOSOME, KEGG_PROTEASOME, and KEGG_HUNTINGTONS_DISEASE, suggesting a possible link to intracellular degradation and neuroimmune modulation (Figure 15A,B).

Together, these GSEA results support that MPO is primarily involved in inflammatory signaling, while TREM2 is associated with structural and stress response pathways, reflecting their complementary yet distinct roles in DFU pathogenesis and impaired wound healing.

#### 3.8.2. Gene Co-Expression Network Analysis of Hub Genes

As shown in Figure 15C, the hub genes were tightly associated with several functionally related genes such as TYROBP, PTGS1, EPX, and ELANE. These interacting partners are enriched in pathways associated with immune activation, oxidative stress, and inflammatory signaling—processes highly relevant to the pathogenesis of diabetic foot ulcers. Notably, MPO and TREM2 were both centrally located within the network, suggesting their potential roles as regulatory nodes mediating leukocyte recruitment and tissue injury responses in DFU. This integrative analysis further supports the pathophysiological relevance of MPO and TREM2 in impaired wound healing.

#### 3.8.3. ceRNA Regulatory Network of TREM2

To explore the upstream non-coding RNA–mediated regulation of the validated hub gene TREM2, a competing endogenous RNA (ceRNA) network was constructed. Initially, no reliable interaction results were obtained from NetworkAnalyst for MPO or TREM2. Therefore, the RNAInter database (http://www.rnainter.org/, accessed on 10 April 2025) was used to predict interactions between hub genes and non-coding RNAs. Interaction pairs with a confidence score greater than 0.7 were selected.

The resulting network revealed that TREM2 is potentially regulated by the miRNA hsa-miR-34a-5p, which in turn is targeted by multiple long non-coding RNAs (lncRNAs). A total of 22 nodes and 22 edges were visualized using Cytoscape (v3.8.2), including 1 hub gene (TREM2), 1 miRNA, and 20 lncRNAs (Figure 15D). Key lncRNAs in this network include well-characterized molecules such as XIST, MALAT1, NEAT1, TUG1, HOTAIR, and MIAT, many of which are known to participate in inflammation, apoptosis regulation, and wound repair processes.

This predicted TREM2–miR-34a-5p–lncRNA axis suggests a potential ceRNA mechanism through which TREM2 expression may be post-transcriptionally regulated in the DFU microenvironment, further emphasizing its centrality in immune modulation and keratinocyte dysfunction.

### 3.9. Clinical Sample Experimental Validation

**Figure 16:**
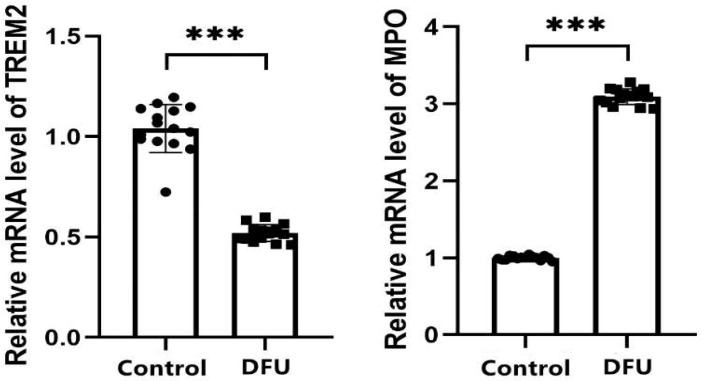
qRT-PCR validation of TREM2 and MPO in ulcer tissue (DFU) and adjacent non-ulcerated skin tissue (control). Mean ± SD. *** *p* < 0.001 vs. control.

The qRT-PCR results showed that TREM2 were underexpressed in the DFU group, while MPO were overexpressed in the DFU group, which was consistent with the bioinformatics analysis. The expression differences were statistically significant (*p* < 0.05) (Figure 16).

## 4. Discussion

DFUs arise from a nexus of neuropathic, ischemic, infectious and immune dysfunctions that lock the wound into a chronic inflammatory state. The clinical burden is severe: rates of amputation and five-year mortality are comparable to those of many malignancies [16]. Despite decades of research, few biomarkers reliably forecast wound trajectory. Recognizing this gap, we interrogated secretory proteins across bulk and single-cell transcriptomic datasets to identify molecules that not only correlate with DFU chronicity but also mediate the intercellular cross-talk driving pathology [17].

Single-cell RNA-sequencing proved particularly powerful in this regard. Chronic wounds comprise a mosaic of keratinocytes, fibroblasts, endothelial cells and diverse immune populations whose responses to hyperglycemia and infection vary [18]. Traditional bulk analyses mask these cell-type-specific signals, whereas single-cell profiling allowed us to map secretory gene expression back to individual lineages. Our data showed that, although these biomarkers are detectable in multiple cell types, DFU-associated differential expression is most pronounced in keratinocytes, which is consistent with the findings of several previous studies. During the re-epithelialization phase of diabetic foot ulcers, keratinocytes migrate from the wound margin to the surface and proliferate to form new epidermis. However, chronic inflammation is the primary obstacle to this process. In diabetic foot ulcers (DFU), the release of IL-1β and TNF-α by numerous M1 macrophages inhibits keratinocyte migration, thereby delaying wound healing. Studies have demonstrated that D-mannose maintains keratinocyte function by inhibiting AGEs formation and activating the AMPK/Nrf2/HO1 pathway, thereby reducing apoptosis and inflammation while restoring cell migration and differentiation [19]. Furthermore, D-mannose-treated keratinocytes inhibit macrophage M1 polarization and promote fibroblast proliferation, collectively enhancing diabetic wound healing [20].

Among the secretory proteins highlighted by our integrative analysis, MPO emerged as a marker of persistent inflammation. MPO is a neutrophil-derived peroxidase that generates hypochlorous acid and is abundant in neutrophil extracellular traps (NETs). MPO is a key enzyme in neutrophils, producing strong oxidants like hypochlorous acid (HOCl) to kill pathogens and clear necrotic tissue. During acute inflammation or early wound healing, neutrophil hyperactivity and elevated MPO levels are essential. In our datasets, MPO expression was markedly declined, particularly within DFU keratinocyte clusters, suggesting sustained neutrophil signaling and oxidative stress in the wound. Although keratinocytes do not directly express MPO, the two are closely associated in wound healing and inflammatory responses. In metabolic diseases such as diabetes, neutrophils often exhibit functional impairments (e.g., reduced chemotaxis, phagocytosis, and activation), which may lead to decreased MPO activity. This is linked to the chronic inflammation and delayed healing observed in diabetic foot [21]. The role of keratinocytes in wound healing extends beyond barrier restoration, as they recruit immune cells (e.g., neutrophils) by secreting cytokines and chemokines. Keratinocytes recruit neutrophils through chemokine secretion (e.g., IL-8) and produce cytokines that promote angiogenesis and matrix remodeling during wound healing. However, reduced MPO levels may impair keratinocyte re-epithelialization, as insufficient MPO compromises effective immune debridement and pathogen clearance, leading to impaired keratinocyte function. Concurrently, restricted neutrophil activity and decreased MPO levels prevent keratinocytes from receiving effective immune regulatory signals, ultimately obstructing the wound healing process [22]. These observations position MPO as both a readout of neutrophil activity and a potential effector of tissue damage in DFUs [23].

TREM2 (Triggering Receptor Expressed on Myeloid Cells 2) is a receptor expressed on the surface of various immune cells, playing an important role in immune response, inflammation regulation, and tissue repair. Although TREM2 is closely associated with macrophages, its role in other cell types, especially keratinocytes, is also worth exploring [24]. Studies suggest that TREM2 may indirectly affect the function of keratinocytes by participating in local inflammatory responses and regulating the immune microenvironment. In diabetic foot ulcers (DFU), the expression of TREM2 may influence the proliferation and migration abilities of keratinocytes [25]. Specifically, TREM2 may regulate local inflammatory responses by influencing the secretion of cytokines from keratinocytes (e.g., IL-6, IL-1β), thereby affecting the wound healing process. For example, TREM2 may suppress excessive inflammation by interacting with its ligands, promoting keratinocyte migration to the wound area, and thus accelerating wound healing. In contrast, dysfunction of TREM2 may lead to the persistence of chronic inflammation, thereby exacerbating the healing impairments in DFU. Furthermore, one study indicated that TREM2 could be detected in the dermal ECM and in keratinocytes/fibroblasts in human skin, but the conclusion regarding the effects on keratinocyte migration/proliferation was that it “may inhibit proliferation and has no significant overall effect on migration” [26]. These data suggest that TREM2 may have the potential to be expressed in epithelial cell populations, but its biological function in keratinocytes has yet to be systematically clarified. In the context of DFU, where keratinocyte dysfunction occurs [27], TREM2 might act as a receptor for epithelial cells to sense the microenvironment (such as advanced glycation end products, high oxidative stress, and matrix damage signals), regulating keratinocyte responses that influence migration or proliferation [27]. At the same time, TREM2 in keratinocytes may regulate inflammation-related signaling pathways (such as inhibiting MAPK/AP-1 or activating PI3K/AKT), improving or suppressing chronic inflammatory microenvironments, and thus indirectly enhancing keratinocyte repair capacity [28]. Although this pathway has not yet been confirmed in keratinocytes, the IL-4/TREM2 axis has been used in macrophage systems to promote wound healing in diabetic models [29]. Furthermore, from the perspective of the “extracellular matrix–keratinocyte–immune cell” triad, if TREM2 is differentially expressed in keratinocytes, it may strengthen the signaling communication between keratinocytes and immune cells (such as macrophages and dendritic cells), thereby improving the re-epithelialization process. Related single-cell studies have also suggested that the failure of keratinocyte migration in DFU is associated with dysregulated immune responses [30]. Of course, there is a clear research gap. On one hand, SCI articles specifically targeting the expression, downstream signaling, and functional validation of TREM2 in keratinocytes are extremely scarce [31]; on the other hand, there have been almost no studies validating the role of TREM2 in keratinocytes in DFU models.

Beyond identifying individual biomarkers, our study explored the regulatory landscape governing these genes, particularly through competitive endogenous RNA (ceRNA) networks. In DFUs, post-transcriptional regulation by non-coding RNAs (like microRNAs, long non-coding RNAs, and circular RNAs) is increasingly recognized as an important layer of control in wound-healing processes [17]. ceRNA networks describe how certain lncRNAs or circRNAs can “sponge” microRNAs, thereby freeing target mRNAs from microRNA suppression. This mechanism can tune the expression of key genes involved in healing. Our analysis indicates that TREM2, for example, might be modulated by multiple miRNAs and lncRNAs, forming a complex regulatory network. This is consistent with previous reports that in DFU, critical signaling genes are embedded in ceRNA interactions. Wang et al. identified a circular RNA, circ_0084443, that is upregulated in DFU tissues and found that it impairs keratinocyte migration and proliferation by sponging a microRNA (acting as a ceRNA) [32,33]. Such dysregulation of keratinocyte function via a ceRNA mechanism can directly contribute to delayed re-epithelialization. Similarly, Liao et al. constructed a DFU-specific ceRNA network comprising several circRNAs, miRNAs, and mRNAs; their analysis highlighted hub genes like BCL2, CCND1, and SMAD4 as potential diagnostic biomarkers in DFU [34]. These findings illustrate that ceRNA networks can influence both cell survival/apoptosis pathways (BCL2), cell cycle progression (CCND1), and growth factor signaling (SMAD4), all of which are relevant to wound healing [35,36,37,38]. In our study, the identification of ceRNA interactions for TREM2 and MPO provides insight into upstream regulators: for instance, we found that TREM2 is a predicted target of multiple miRNAs (via databases like miRWalk and TargetScan), suggesting that overexpression of certain DFU-upregulated miRNAs could be suppressing TREM2 expression [39,40]. Likewise, MPO—while primarily controlled at the level of myeloid cell activation—may also be subject to post-transcriptional control by miRNAs or RNA-binding proteins. Understanding these networks is more than an academic exercise; it opens new avenues for intervention [41,42]. If a particular non-coding RNA is keeping a pro-healing gene repressed, targeting that ncRNA (with an antisense oligonucleotide or siRNA) could release the brake on healing. For example, if an lncRNA were found to sponge a miRNA that normally suppresses a pro-angiogenic factor, inhibiting that lncRNA might restore angiogenesis in the wound. Our findings lay the groundwork for such hypotheses. They also reinforce that DFU pathology is not only about protein-coding genes but also involves a mis-regulation of the non-coding genome [43]. Future research integrating transcriptomic data with epigenetic and non-coding RNA analyses will be important to fully elucidate these regulatory circuits. Ultimately, therapies modulating ceRNA network components (e.g., using miRNA mimics/inhibitors or decoy targets) could complement protein-centric therapies to rebalance the wound healing process [44].

The translational implications of our findings are manifold. First, measuring secretory protein levels in wound exudate or tissue could aid risk stratification: persistent elevation of MPO may herald an ulcer mired in an inflammatory state, whereas low TREM2 might indicate failure to transition to repair. Elevated NET biomarkers containing MPO-bound DNA have already been linked to poor DFU outcomes [22]. Second, these molecules themselves are actionable. Limiting MPO activity is attractive; approaches include blocking neutrophil recruitment, inhibiting NETosis or scavenging the oxidants produced. Notably, the repurposed drug disulfiram has been shown to suppress NET formation via NLRP3 inflammasome and gasdermin-D inhibition, thereby accelerating wound closure in diabetic mice [45]. Conversely, augmenting TREM2 signaling could push macrophages toward a reparative phenotype. Administration of soluble TREM2 protein ameliorated diabetic wound healing and dampened inflammation in preclinical models [32], and pharmacological stabilization of downstream pathways may replicate these effects [32]. Combination strategies that simultaneously curb destructive neutrophil responses and enhance macrophage-mediated repair merit exploration. Finally, other candidates from our panel warrant attention: LYZ, a secreted antimicrobial enzyme, may reflect microbial burden and could inform antimicrobial therapy, whereas the transporter SLCO2B1 could influence prostaglandin or nutrient flux. Given that a related transporter, SLCO2A1, modulates prostaglandin E2 levels and its inhibition improves healing [46], investigating SLCO2B1 may reveal new metabolic leverage points. Together, these insights lay a foundation for diagnostic panels and targeted therapeutics tailored to the molecular phenotype of each DFU. In the clinical DFU samples, the PCR results showed an upregulation of MPO, despite bioinformatic analyses indicating a downregulation of both MPO and TREM2. This discrepancy could be attributed to the fact that the samples we collected may have been during an infectious phase, when neutrophil infiltration is typically elevated. During infection, MPO, which is predominantly expressed by neutrophils, is often upregulated as part of the acute inflammatory response. This could explain the higher MPO expression observed in our PCR experiments, highlighting the dynamic nature of the inflammatory processes in DFUs.

Like all computational studies, ours has limitations. Although all discovery analyses (DEG, WGCNA and machine learning) were deliberately confined to a single-platform training cohort to avoid cross-platform batch effects, heterogeneity between independent cohorts—including differences in platform, sampling procedures and patient characteristics—may still influence effect sizes and diagnostic performance. Second, we validated biomarkers in separately normalized external cohorts rather than conducting a fully integrated batch-corrected meta-analysis; therefore, prospective multi-centre studies will be required to further confirm the generalizability of MPO and TREM2. Our conclusions are based primarily on transcriptomic patterns; future work must evaluate protein abundance and enzymatic activity in situ to confirm functional relevance. Mechanistic experiments, such as gene manipulation in keratinocytes or macrophages and testing MPO or TREM2 modulation in diabetic wound models, are needed to establish causality. Patient heterogeneity—including variations in age, comorbidities and ulcer duration—could influence gene expression; larger, prospective cohorts and longitudinal sampling will help generalize our findings. The ceRNA relationships predicted here require validation through as-says of miRNA–lncRNA–mRNA interactions. Our ceRNA analysis is purely computational. The inferred lncRNA–miRNA–MPO/TREM2 axes were derived from interaction databases and co-expression patterns, and thus are hypothesis-generating only. We did not perform reporter assays or loss-/gain-of-function experiments to validate these interactions; therefore, no causal conclusions can be drawn at this stage, and future functional studies are required to determine whether these predicted ceRNA relationships truly modulate MPO and TREM2 in DFU. Investigating upstream triggers, such as hypoxia, advanced glycation end-products and biofilm-derived signals, may reveal why secretory protein signaling is dysregulated in DFUs. Addressing these questions will be critical for translating secretory protein biomarkers into precision diagnostics and therapeutics for patients with diabetic foot ulcers.

## Data Availability

Data is contained within the article.

## Supplementary Materials

The following supporting information can be downloaded at: https://www.mdpi.com/article/10.3390/genes16121419/s1, Table S1: cDNA reaction system, Table S2: cDNA reaction conditions, Table S3: qPCR reaction system, Table S4: qPCR amplification conditions, Table S5: Primer sequences.
