# Supplementary material for: Integrated Multi-Omics and Independent Validation Reveal MPO and TREM2 as Secretory Biomarkers for Non-Healing Diabetic Foot Ulcers
Source: Genes (Basel). 2025 Nov 28;16(12):1419. doi: 10.3390/genes16121419 (PMC12732572; doi:10.3390/genes16121419)
Supplement: Supplementary file 1 [file genes-16-01419-s001.zip › genes-3999854-supplementary.pdf]

Supplementary Table S1. cDNA reaction system.

Component	Volume
GoScript™ Enzyme Mix	4ul
GoScript™ Reaction Buffer	4ul
Total RNA	2ug
Nuclease-Free Water	Add to 20ul

Supplementary Table S2. cDNA reaction conditions.

Temperature	Time
25℃	5min
42℃	60min
70℃	15min
4℃	hold

Supplementary Table S3. qPCR reaction system.

Component	Volume
cDNA	1ul
2xUniversal Blue SYBR Green qPCR Master Mix	5ul
Forward primer (10μM)	0.25ul
Reverse primer (10μM)	0.25ul
ddH_2O	5.5ul

Supplementary Table S4. qPCR amplification conditions.

	Temperature	Time
Initial Denaturation	95℃	5min
Denaturation	95℃	30s
Annealing and Extension	60℃	60s

Supplementary Table S5. Primer sequences.

Primer	Sequence
MPO F	GAGCAGGACAAATACCGCACCA
MPO R	AGAGAAGCCGTCCTCATACTCC
TREM2 F	ATGATGCGGGTCTCTACCAGTG
TREM2 R	GCATCCTCGAAGCTCTCAGACT
GAPDH F	GTCTCCTCTGACTTCAACAGCG
GAPDH R	ACCACCCTGTTGCTGTAGCCAA
